# Adenovirus infection is associated with altered gut microbial communities in a non-human primate

**DOI:** 10.1038/s41598-019-49829-z

**Published:** 2019-09-16

**Authors:** Wasimuddin, Victor M. Corman, Jörg U. Ganzhorn, Jacques Rakotondranary, Yedidya R. Ratovonamana, Christian Drosten, Simone Sommer

**Affiliations:** 1 University of Ulm, Institute of Evolutionary Ecology and Conservation Genomics, Ulm, Germany; 2 Institute of Virology, Charité-Universitätsmedizin Berlin, Corporate member of Free University, Humboldt-University and Berlin Institute of Health, Berlin, Germany; 3 German Centre for Infection Research (DZIF), Berlin, Germany; 4 Institute of Zoology, Dept. Animal Ecology and Conservation, Universität Hamburg, Hamburg, Germany; 5 Département Biologie Animale, Faculté des Sciences, P.O Box 906, Université d’Antananarivo, Antananarivo, 101 Madagascar; 6 Département de Biologie et Ecologie Végétale, Faculté des Sciences, P.O Box 906, Université d’Antananarivo, Antananarivo, 101 Madagascar

**Keywords:** Bacteria, Microbial communities

## Abstract

Adenovirus (AdV) infections are one of the main causes of diarrhea in young children. Enteric AdVs probably disrupt gut microbial defences, which can result in diarrhea. To understand the role of the gut microbiome in AdV-induced pathologies, we investigated the gut microbiome of a naturally AdV-infected non-human primate species, the Malagasy mouse lemur (*Microcebus griseorufus*), which represents an important model in understanding the evolution of diseases. We observed that AdV infection is associated with disruption of the gut microbial community composition. In AdV+ lemurs, several commensal taxa essential for a healthy gut microbiome decreased, whereas genera containing potential pathogens, such as *Neisseria*, increased in abundance. Microbial co-occurrence networks revealed a loss of important microbial community interactions in AdV+ lemurs and an overrepresentation of Prevotellaceae. The observation of enteric virus-associated loss of commensal bacteria and associated shifts towards pathobionts may represent the missing link for a better understanding of AdV-induced effects in humans, and also for their potential as drivers of co-infections, an area of research that has been largely neglected so far.

## Introduction

Adenoviruses (AdVs) are non-enveloped, icosahedral, double-stranded DNA viruses commonly found in vertebrate host species including fish, amphibians, reptiles, birds, and mammals^1^. In humans and other primates, AdVs can cause mild to severe diseases, such as respiratory infections, epidemic conjunctivitis, and gastroenteritis^2,3^. They also cause severe diarrhea in children and are major viral pathogens in immunocompromised adults, associated with high morbidities and mortalities in both age groups^2,4,5^. Infections are typically caused by the inhalation of aerosolized droplets from an infected host, by direct conjunctival inoculation, or via faecal-oral transmission^2^. AdVs commonly persist in the whole gastrointestinal tract of infected individuals and the detection of viral loads in stools provides an early measurement of AdV disease^2^. AdV-induced lesions in gut enterocytes together with the deterioration of villi and compensatory hyperplasia in crypts have all been proposed to explain the observed gut malabsorption and loss of fluids after AdV infections^6^.

The gastrointestinal tract contains a myriad of densely populated, commensal bacteria: the so-called gut microbiome. The gut bacterial community provides essential nutritional services to its host, is an important driver of mucosal immunity, and provides protection against enteric pathogens^7^. It maintains the homeostasis of the gastrointestinal tract and regulates intestinal epithelial cell turnover, the promotion of epithelial restoration, and the re-organization of tight junctions, all of which are critical for maintaining the gut-barrier function^8,9^. The AdV-induced disruption of epithelial cells might indirectly result in disturbance of the gut microbiome homeostasis, leading or contributing to diarrhoea, as experienced during viral gastroenteritis^10^. Previous work has shown that different viral infections are linked to alterations and disruptions of the intestinal microbiome^11–15^. Yet, this area of research is still underdeveloped and specifically studies aiming to resolve the causes and sequelae of AdV induced gastroenteritis and the role of associated altered gut microbiomes are lacking.

In humans, children exhibit a higher AdV prevalence than adults. The higher AdV susceptibility of younger individuals has been explained on the basis of their immature immune system^2,16^. The higher disease resilience of adults has also been suggested to be the result of a protective immunity, attributable to previous AdV exposure during childhood^2^. However, the diversity and composition of the microbiome is known to change with host age, a finding that, in turn, has been shown to influence susceptibility to enteric pathogens^17^. Investigations of the gut microbiome might therefore contribute to our understanding of AdV disease pathology and, particularly, to the development of severe diarrhea in children. Indeed, it has recently been shown in bats that Astrovirus infections, that may cause (like AdV) severe diarrhea in humans, can perturb the gut microbiome and induce age-dependent dysbiosis^14^. Despite these important potential links, no study has so far investigated the influence of AdV on the gut microbial community and potentially age-related AdV pathologies.

Non-human primates provide natural model systems for exploring potential AdV-gut microbiome interactions. Non-human primates are increasingly being implicated as potential sources of zoonotic diseases, including AdVs, in humans^18–20^. A high AdV strain diversity has been observed in non-human primates and, notably, many of the non-human primate AdV strains show close sequence similarity to human AdVs^20,21^. Interspecies transmission events between humans and other primates have been postulated for some AdV strains and are becoming increasingly evident^18,20,22,23^. Malagasy mouse lemurs are of intense interest in research to understand the convergent evolution of diseases^24^. Initial evidence of AdV-infection of Malagasy lemurs, at surprisingly high frequencies, has recently been presented^25^. The lemurs’ home ranges overlap with those of other endangered species and, since they also occur in human-modified habitats, they come into potential contact with domestic animals and humans^25^. As AdVs are one of the common causes of gastroenteritis in humans in Madagascar, the potential for co-circulation of AdVs between humans and lemurs has been suggested^25^. Malagasy lemurs thus provide a natural study scenario for interactions between AdV infections and the gut microbiome that can provide important insight into mechanisms of disease in humans.

In this study, we have tested whether naturally occurring AdV infections influence the gut microbiome of free-living mouse lemurs (*Microcebus griseorufus*; primates of Madagascar). Given the higher AdV disease severity in children, we tested for age-dependent effects by comparing young and adult, infected and uninfected lemurs. If AdV-infections disturb the gut bacterial homeostasis, resulting in the loss of commensal bacteria and/or increase in pathobionts, this could promote co-infections and possibly increase previously suppressed health risks.

## Results

We measured the impact of AdV infection on the gut microbiome of an endemic non-human primate species, the grey-brown mouse lemur (*M. griseorufus*). We characterized the gut microbiome of 160 individuals (44 AdV-positive samples: 18 young, 19 adults; 116 AdV-negative samples: 43 young, 66 adults; for 14 individuals age could not be determined) by performing high throughput sequencing of the V4 region of the bacterial 16S ribosomal RNA gene. We recovered, on average, 56,216 (range 22,248–148,060) high quality reads per sample after taxonomic assignments. The gut bacterial community of the mouse lemur is dominated by the phyla Actinobacteria (34.4%), Bacteroidetes (28.4%) and Firmicutes (26.2%), followed by Proteobacteria (9.2%) and Cyanobacteria (0.6%).

### Adenovirus infection is associated with altered gut microbial communities in lemurs

We used General Linear Modelling (GLM) to test whether inter-individual differences within three alpha diversity estimates (number of observed species (OTUs), Chao1 and phylogenetic diversity (PD)) could be explained by AdV infection, host age and the AdV*age interaction. There was no effect of AdV and the AdV*age interaction (all >0.05) on all tested alpha diversity indices. Alpha diversity, as measured by the number of observed species (p = 0.002), Chao1 (p = 0.007) and PD (p = 0.003) was lower in young lemurs compared to adults (Supplementary Table 1, Supplementary Fig. 1).

**Figure 1:**
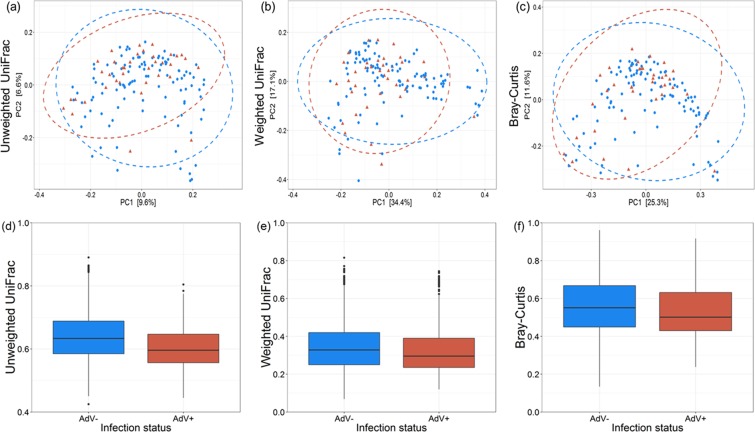
AdV infection is associated with altered gut bacterial community composition of mouse lemurs. Principal-coordinate plots of (**a**) unweighted UniFrac (p = 0.016), (**b**) weighted UniFrac (p = 0.055) and (**c**) Bray-Curtis (p = 0.037) metrics in mouse lemurs. Dots and surrounding dashed ellipses (95% confidence level) represent gut bacterial communities of AdV− (blue) and AdV+ (red) individuals. Boxplots are showing the inter-individual distances between AdV− (blue) and AdV+ (red) individuals using (**d**) unweighted UniFrac, (**e**) weighted UniFrac and (**f**) Bray-Curtis metrics in mouse lemurs (all < 0.001).

To determine, whether AdV infections influence the gut microbial community composition, we calculated three beta diversity metrics (unweighted UniFrac, weighted UniFrac and Bray-Curtis) and included again AdV infection, host age and AdV*age interaction as explanatory variables in PERMANOVA models. We observed a significant effect of AdV infection (unweighted UniFrac: R2 = 0.010, p = 0.016; weighted UniFrac: R2 = 0.013, p = 0.055; Bray-Curtis: R2 = 0.013, p = 0.037) and host age (unweighted UniFrac: R2 = 0.017, p = 0.001; weighted UniFrac: R2 = 0.031, p = 0.005; Bray-Curtis: R2 = 0.029, p = 0.001), on gut microbial beta diversity estimates (Fig. 1, Supplementary Fig. 2, Supplementary Table S2). However, no effect of the AdV*age interaction (all > 0.05) on the gut microbial community composition was observed (Supplementary Table S2, Supplementary Fig. 3). Overall inter-individual beta diversity significantly decreased in AdV-infected compared to uninfected lemurs (unweighted UniFrac; weighted UniFrac; Bray-Curtis; all p < 0.001) (Fig. 1).

The average body mass of AdV+ individuals tended to be lower than of AdV− individuals (p = 0.062, Supplementary Fig. 4). However, an individual’s body mass showed no influence on bacterial alpha diversity (number of observed species (p = 0.140), on Chao1 (p = 0.157) and on PD (p = 0.164)) or community composition (unweighted UniFrac: R2 = 0.007, p = 0.193; weighted UniFrac: R2 = 0.009, p = 0.194; Bray-Curtis: R2 = 0.008, p = 0.176).

In order to investigate the observed changes in the community composition of AdV-infected individuals further and to understand the influence of AdV infection, host age and AdV*age interaction, we performed similar PERMANOVA models on the five major phyla (Actinobacteria (34.4%), Bacteroidetes (28.4%), Firmicutes (26.2%), Proteobacteria (9.2%) and Cyanobacteria (0.6%)). The unweighted UniFrac distances in Actinobacteria (unweighted UniFrac: R2 = 0.010, p = 0.016) and Bacteroidetes (unweighted UniFrac: R2 = 0.011, p = 0.035), as well as the weighted UniFrac distances of Proteobacteria (weighted UniFrac: R2 = 0.019, p = 0.016) were lower in AdV+ individuals (Supplementary Fig. 5).

### AdV infection is associated with shifts in the relative abundance of major taxa and OTUs

**Figure 2:**
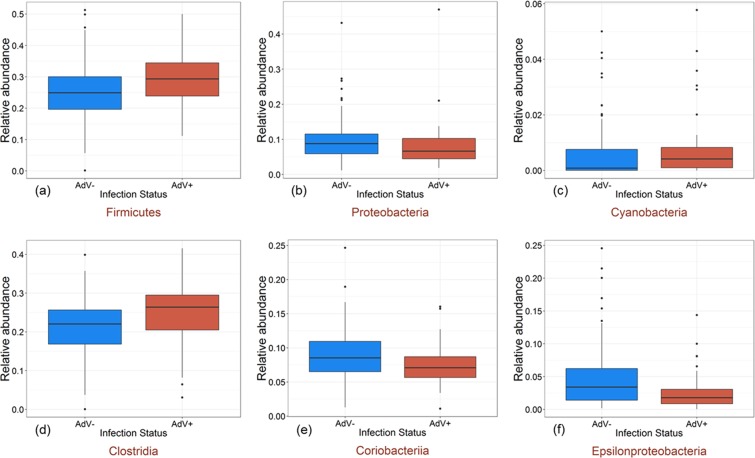
Relative abundance of major phyla and classes change in relation to AdV infection. Box plots indicate the relative abundance of major phyla (**a–c**) and classes (**d–f**) in the gut microbiomes of AdV− (blue) and AdV+ (red) mouse lemurs (phylum: Firmicutes (p < 0.001), Proteobacteria (p = 0.041), Cyanobacteria (p = 0.012); class: Clostridia (p = 0.008), Coriobacteriia (p = 0.021), Epsilonproteobacteria (p = 0.006)).

AdV-infections differentially influenced the relative abundance of taxa at higher taxonomic levels. Among the five major phyla, we detected an increase in the relative abundance of Firmicutes (p < 0.001) and Cyanobacteria (p = 0.012) whereas the relative abundance of Proteobacteria (p = 0.041) decreased in AdV-infected individuals (Fig. 2). At the class level, we observed an increase in Clostridia (p = 0.008) but a decrease in Coriobacteriia (p = 0.021) and Epsilonproteobacteria (p = 0.006) in AdV+ individuals (Fig. 2). None of the five phyla showed significant shift in their relative abundance according to age (all >0.05). At the class level, however, the relative abundance of Epsilonproteobacteria was lower in adults compared to young lemurs (p = 0.002). In order to identify those OTUs that differed in their abundance because of AdV infection, we employed negative binomial-based Exact tests^26^. We identified 78 OTUs in mouse lemurs that differed significantly (p < 0.01) in abundance, with 51 OTUs (65.3%) revealing a lower and 27 OTUs (34.6%) revealing a higher mean abundance in AdV+ compared to AdV- individuals (Supplementary Table S3). Twenty OTUs were completely absent in AdV+ mouse lemurs (Supplementary Table S3).

**Figure 3:**
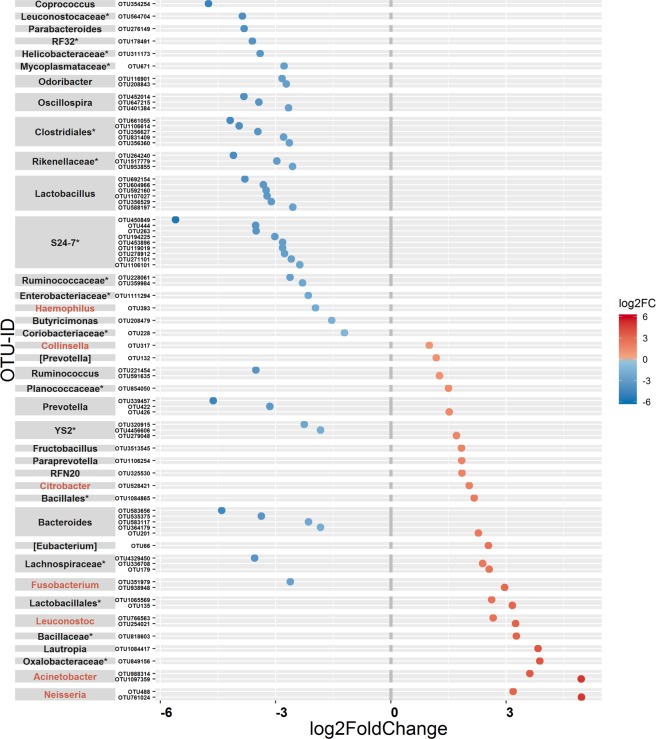
Differential abundance of OTUs in relation to AdV infection in mouse lemurs. Shown are OTUs (78 OTUs) that differ in their mean abundance (between AdV− and AdV+ category) in relation to the infection status of mouse lemurs. OTUs are arranged according to increasing values of log_2_-fold change and grouped by their respective genus. The values indicate a log_2_-fold decrease (blue dots, 51 OTUs) or increase (red dots, 27 OTUs) in OTU abundance in AdV infected individuals compared to uninfected individuals (see Table S3 for more details). The highest possible taxonomic assignment (maximal to the genus level) is shown next to each OTU and potential pathogenic genera are marked in red. *Includes unclassified OTUs at genus level.

Picked OTUs that showed a lower abundance in AdV+ mouse lemurs mainly belonged to the genera *Lactobacillus* (n = 6 OTU), *Bacteroides* (n = 4 OTU), and *Oscillospira* (n = 3 OTU) and to the families S24–7 (n = 9 OTU), Ruminococcaceae (n = 6 OTU) and Lactobacillaceae (n = 6 OTU) (Fig. 3, Supplementary Table S3). OTUs that showed a higher abundance in AdV+ individuals mainly belonged to the potential pathogenic genera *Neisseria* (n = 2 OTU), *Acinetobacter* (n = 2 OTU), *Leuconostoc* (n = 2 OTU) and *Citrobacter* (n = 1 OTU) (Fig. 3, Supplementary Table S3).

### Microbial networks differ between AdV− and AdV+ lemurs

**Figure 4:**
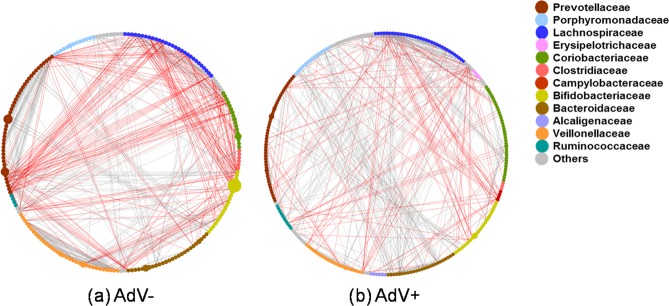
Microbial networks of OTUs in relation to AdV-infection in mouse lemurs. Shown are the networks of (**a**) AdV− and (**b**) AdV+ mouse lemurs (sub networks are not shown). Nodes representing OTUs are coloured based on their corresponding families (in inset) and node sizes are according to the relative proportion. Red edges indicate negative relationships and grey edges positive relationships between the interacting OTUs. Both, positive (AdV− = 1003; AdV+ = 974) and negative (AdV− = 229; AdV+ = 142) interactions decrease in the AdV+ network compared to the AdV− network.

We constructed microbial co-occurrence networks to determine the relationships among the OTUs observed in AdV− and AdV+ individuals, respectively. In the networks, nodes represent OTUs and node sizes are according to the relative proportion of specific OTUs (Fig. 4). Negative and positive relationships were shown as edges between the interacting OTUs (Fig. 4). In AdV− mouse lemurs, the network consisted of 414 nodes and 1232 edges, whereas the AdV+ network consisted of 454 nodes and 1116 edges (Fig. 4), thus the number of edges per node was lower in AdV+ (2.46) compared to AdV− (2.97) individuals. This was mainly due to the lower number of negative interactions (negative edges: AdV−: 229, AdV+: 142 (=38% decrease) in the AdV+ network (Fig. 4). The AdV infection mostly affected the number of negative interactions related to the family Lachnospiraceae, most of which got lost in AdV+ individuals (Fig. 4). However, few members of Campylobacteraceae showed only negative interactions with various other OTUs in the AdV+ network.

We also constructed the microbial network based on the OTUs that differed significantly in abundance between AdV− and AdV+ individuals (as identified by the Exact-test approach, i.e. 78 OTUs, see above). The network consists of 47 nodes and 146 edges (Supplementary Fig. 6). OTUs which decreased in abundance after AdV infection showed positive interactions with each other, whereas OTUs which increased in AdV+ individuals showed a negative interaction with those who decreased in abundance. Similar to the main network, here OTUs of *Prevotella* revealed negative interactions with several other OTUs, including member of potential pathogenic genus *Neisseria* (OTU488) which increased in abundance in AdV+ individuals (Supplementary Fig. 6).

## Discussion

AdVs are one of the main causes of childhood diarrhea and serious infections, especially in immunocompromised adults, leading to their association with severe morbidity and mortality^2^. The gut microbiome has emerged as a critical player in maintaining gut immunity and, in recent years, evidence has accumulated showing that virus infections induce gut microbiome dysbiosis^11–15^. By utilizing a naturally AdV-infected non-human primate model, the Malagasy grey-brown mouse lemur, we have examined, for the first time, AdV-gut microbiome interactions. Our study has revealed that AdV infections are indeed associated with disturbances of the gut microbiome. Although AdV infections had no impact on the alpha diversity, the beta diversity was changed in AdV+ individuals. This is similar to observations of the gut microbiomes in chimpanzees following simian immunodeficiency virus (SIV) infections^11^. In AdV-infected lemurs, the beta diversity decreased because of predominant depletion of essential bacteria taxa, and an increase in abundance of potentially pathogenic bacterial taxa.

Shifts in bacterial diversity beyond the ‘normal’ range can result in dysbiosis, e.g. the depletion in bacteria essential for host health and an increase in pathogenic bacteria. Both can have negative consequences on host immunity and health. Gut microbial community disruption can cause diarrhea not only directly^27^, but also indirectly by driving imbalance in gut homeostasis, leading to increased susceptibility towards diarrhea-promoting pathogens^28,29^. An increase in beta diversity has frequently been reported in individuals infected with pathogens that interfere with the host immune system, such as HIV^30^ or SIV^11^, or in other diseases such as inflammatory bowel disease (IBD)^31^. In contrast, fewer reports are available showing a decrease in beta diversity associated with viral infections^14,32^.

An increase in beta diversity after dysbiosis was previously explained using the ‘Anna Karenina principle’ (AKP) for animal microbiomes, stating that ‘dysbiotic individuals vary more in microbial community composition than healthy individuals - paralleling Leo Tolstoy’s dictum that all happy families are alike; each unhappy family is unhappy in its own way’^33^. According to Zaneveld *et al*.^33^, under mild stress conditions, individuals differ in their stress response and not all individuals show shifts to new bacterial community composition, resulting in an increase in beta diversity. Under severe stress conditions, however, all individuals are stressed and show shifts to new compositions which are similar to one another and, as a result, the beta diversity remains similar to that of healthy individuals, although the bacterial composition is dissimilar. Such a differential response of individuals under mild stress conditions is defined as the AKP effect for animal microbiomes. The case of a reduced beta diversity below the healthy level attributable to specific types of stressors is described as Anti-AKP effect^33^. We argue that an Anti-AKP effect could be simply an extreme stage of the AKP effect, rather than the effect of specific stressors, i.e. mild stress leads to an increase but severely stressed conditions lead to a drastic reduction in community composition and thus lower beta diversity compared to that of healthy subjects. Such a scenario has been recently observed in bats, where severe stress due to Astrovirus (AstV) infections in young individuals caused a reduction in beta diversity, whereas mild stress, probably attributable to an improved immune system, resulted in an increase in beta diversity in adult individuals^14^. However, in the case of AdV infected mouse lemurs, we did not observe differential age-dependent effects of AdV infections on beta diversity, but an overall reduction in beta diversity, suggesting a severe stress condition due to AdV infections independent of age.

Differences in the bacterial community composition between AdV-infected and -uninfected individuals were primarily associated with three major phyla: Actinobacteria, Bacteroidetes, and Proteobacteria. All of these phyla showed a decrease in beta diversity (i.e. UniFrac distances) in AdV+ individuals compared to uninfected mouse lemurs. Actinobacteria and Bacteroidetes have been shown to be involved in colonization resistance against enteric pathogens^34^ and a reduction in their diversity in the gut microbiome as a result of AdV-infections can have negative effects on lemur health.

Associated with shifts in beta diversity, changes in the relative abundance of specific bacterial taxa were observed at various taxonomic levels in AdV+ individuals. At the higher phylum level, Firmicutes and Cynobacteria increased in relative abundance, whereas the relative abundance of Proteobacteria decreased in AdV+ mouse lemurs. An increase in Firmicutes has been found to be associated with obesity in humans as well as in other animals^35–37^. It is interesting to observe that Firmicutes increased in AdV+ lemurs since a recent study reported AdV-induced obesity in humans and proposed microbiome dysbiosis as a potential cause^38^. A decrease in Proteobacteria was also previously observed in AstV-infected bats^14^, however, an increase in Proteobacteria is suggested as potential signature of gut microbial dysbiosis^39^. At the class level, an increase in Clostridia and decrease in Coriobacteriia and Epsilonproteobacteria were observed in AdV+ individuals. The class Clostridia contains both beneficial and pathogenic bacterial taxa^40^ and its increased abundance has been shown in diseases such as gastric cancer^41^ and especially obesity^42^. Likewise, members of Coriobacteriia contain both beneficial bacteria as well as potential pathogens. Coriobacteriia has been shown to decrease in abundance during chronic inflammatory diseases^43^ and has recently been suggested as a therapeutic taxon against type 2 diabetes^44^. It has been postulated that commensal Epsilonproteobacteria provide protection against obesity-associated disorders in primates^45^. Thus, a decrease in Epsilonproteobacteria associated with AdV infection could make individuals vulnerable to obesity^38^. Similarly, a decrease in Epsilonproteobacteria with age could be associated with increasing vulnerability towards obesity with increasing age in human and non-human primates^46^.

Further, we detected changes in bacterial abundances at the OTU level in AdV infected versus uninfected mouse lemurs. OTUs that differed significantly between AdV− and AdV+ individuals mainly showed a decrease in the relative abundance in infected mouse lemurs, however, some OTUs also increased. This corresponds to two major observed trends, namely a decrease in beneficial bacteria and an increase in pathogens, which have been recognized in a recent meta-analysis aiming at detecting a common or disease-specific microbial pattern^47^. The majority of OTUs that showed changes in abundance after AdV infection belonged to the families S24-7, Ruminococcaceae, and Lactobacillaceae, which prominently decreased in abundance in AdV-infected mouse lemurs. Fourteen OTUs from these three families were completely absent in AdV+ mouse lemurs. Members of Ruminococcaceae and Lactobacillaceae families are involved in colonization resistance against pathogens and a reduced abundance is observed in *Clostridium difficile* infection^48^, in diarrhea^49^ among others^47,50^. However, the function of the family S24-7 is largely unknown, as it remains uncultured so far^51^, although an increased abundance of S24-7 has been positively correlated with the remission of colitis^52^ and it is also found to be associated with the hibernation of arctic ground squirrels^53^. Mouse lemurs can go into torpor during the cold dry seasons, when food and water supply is limited^54^. Assuming its potential role in hibernation, a decreased abundance of S24-7 in AdV+ individuals might thus negatively influence mouse lemur physiology during dry seasons. Predominant OTUs from genera showing a decrease in AdV+ individuals (*Lactobacillus*, *Bacteroides*, and *Oscillospira*) are defined as core members of the gut microbiome. Core members are groups of microbes commonly found within a host’s microbiome, which have critical functions and are essential for gut homeostasis^55^. For example, *Lactobacillus, Bacteroides*, and *Oscillospira* are responsible for the production of many short-chain fatty acids (SCFAs), especially butyrate, which not only represent an important source of energy for gut epithelial cells, but more importantly maintain the epithelial barrier integrity. Loss of these SCFA-producers can result in diarrhea^49,56–58^. Several members of *Lactobacillus* are commonly recommended as probiotics and have been extensively used in combatting different diseases^59^. They counteract against pathogens^58^ and often show a reduced abundance after pathogenic infection such as HIV or SIV^60^. *Bacteroides* play an important role in maintaining gut immunity and are reduced in abundance by several diseases, such as IBD^61^. Colonization of germ-free mice with *Bacteroides* can rectify several immune system defects^62^. Similarly, members of the genus *Oscillospira* are important gut commensals and are involved in the digestion of an animal-based diet^63^. *Oscillospira* is associated with leanness in humans and showed decrease in abundance in obesity but also in other diseases^63,64^. Moreover, we observed an increase in abundance of OTUs from potential pathogens, such as *Acinetobacter*, *Citrobacter*, *Leuconostoc*, and *Neisseria*. The genera *Acinetobacter*, *Citrobacter*, and *Leuconostoc* contain known pathogens^65–67^, whose abundance increased due to different diseases, leading to dysbiosis^14,68–71^. Likewise, many species of *Neisseria* are pathogenic and further show an increased abundance in microbiomes of hosts with various gut inflammatory diseases^64,72^. In particular, *N. gonorrhoeae* has not only been found to be a driver of HIV expression, it is also one of the most common sexually transmitted infections in HIV-infected people^73^. However, an OTU assigned to a potentially pathogenic *Haemophilus* genus was also noticed with a lower abundance in AdV+ mouse lemurs.

Our observation of microbial changes associated with viral infections is in agreement with previous studies showing an increased abundance of known pathogenic bacteria in diseases such as irritable bowel syndrome^68^, cancer^71^, and multiple scelerossis^69^. This suggests that viral infections or diseases could lead to a dysbiosis in the gut causing an increase in pathogens. In order to rule out and to disentangle whether an increase in abundance of bacterial pathogens in the gut is not the outcome but primarily the cause of a host’s increased susceptibility towards AdV infections^74^, studies are required that follow healthy individuals and individuals with a disturbed microbiome through time, i.e. pre- and post AdV infections. Thus a longitudinal study or intervention study would be necessary.

Our constructed microbial networks for mouse lemurs’ gut microbiome showed an interesting similarity with the human gut microbiome enterotype-2 network, specifically in over-representation and predominantly negative interactions of the Prevotellaceae family^75^. We observed a high relative abundance of *Prevotella* (13.1%, second most abundant genus) in the microbiome of mouse lemurs, which is comparable to findings concerning human enterotype-2 and also in chimpanzees^76^. High abundance of Prevotellaceae/*Prevotella* is associated with a carbohydrate-rich diet in humans and chimpanzees, which is also true for mouse lemurs. We observed a relatively shallow network in AdV+ compare to AdV− mouse lemur individuals, which was mainly due to a drastic decrease (38%) in negative interactions in the AdV+ network mainly attributable to the Lachnospiraceae family. Members of Lachnospiraceae have been shown to exhibit negative interactions with invading pathogens^14,77^, are important components of gut health, and their loss results in diarrhea^49^. Our microbial network analysis based on those OTUs that were significantly affected by AdV infections revealed a similar *Prevotella*-dominant network as observed for the main network. OTUs which increased in abundance in AdV+ mouse lemurs showed negative interactions with others which decreased in abundance. Members of *Prevotella* showed both an increase and a decrease in abundance and accordingly showed both positive and negative interactions with each other. *Prevotella* (OTU132) also showed negative interaction with potentially pathogenic *Neisseria* (OTU488), due to their shared functional potential as reported earlier^78^.

To conclude, our study reveals significant interactions between AdV, a major cause of infectious diarrhea in humans, and the gut microbiome, an important component of the gut immune defence, utilizing natural AdV infected Malagasy mouse lemurs as a primate representative. We found that AdV are indeed associated with the disruption of gut microbiomes. In AdV+ individuals, the abundance of beneficial gut bacteria was depleted, together with an overall shrinkage of the gut microbiome community composition, and an increase in abundance of potentially pathogenic bacterial taxa. The disturbance of the gut bacterial homeostasis might increase previously suppressed health risks by promoting co-infections. Future research in this area should aim at longitudinal wildlife data in order to better understand temporal changes in the gut microbiome before and after AdV infection and to investigate the role of the immune system in AdV-induced pathologies. Advancement in our understanding of such AdV-induced pathologies might contribute to develop a targeted microbiome-based therapy against AdV infections, especially in childhood, during which this infection still constitutes a prime cause of diarrhea-associated morbidities and mortalities.

## Materials and Methods

### Sample collection and adenovirus testing

To investigate the influence of Adenovirus (AdV) infections on the gut microbiome, we chose an endemic non-human primate species, the grey-brown mouse lemur (*Microcebus griseorufus*). Mouse lemurs offer an interesting model for studying AdV infections because of (1) an assumed high AdV prevalence, (2) a range overlap with other endangered species and (3) potential contact with domestic animals and humans^79,80^. *Microcebus griseorufus* were captured in Sherman live-traps (H.B. Sherman Traps, Tallahassee; 7.5 × 7.5 × 30.5 cm) between 2013 and 2015 in the dry spiny forest in southern Madagascar. All trapped animals were sexed, measured, weighed and marked permanently with subdermal transponders. None of the individuals exhibited any signs of illness or disease at the time of capture. Individuals were aged based on body mass and capture month as sexually active “adults” and sexually non-mature “young”. The reproduction of mouse lemurs is highly seasonal^54^. Offspring are born between January and February and are sexually mature after the hibernation period (ca. July – September). Fresh faecal samples were collected either from the traps or handling bags and preserved in Eppendorf vials filled with 500 µl RNAlater (Life Technologies). Upon return to the field station, samples were kept in a cool place (around 20 °C) until they could be transported to the next city. There, samples were stored at −20 °C, before they were transported in a Styrofoam isolation box equipped with passive cooling elements to Germany where they were kept at −80 °C until DNA extraction.

We chose a Next-generation Sequencing (NGS) approach followed by targeted polymerase chain reaction (PCR) screening to elucidate the occurrence of AdVs in mouse lemurs. The total nucleic acids were extracted from 50 µl of the feces RNAlater suspension of 160 individual lemurs by using the MagNAPure 96 and the Viral NA Small Volume Kit (Roche, Mannheim, Germany) according to the manufacturer’s instructions. For the initial NGS approach, all 160 samples were pooled in groups of 10 samples each. Library construction and Illumina MiSeq sequencing was carried out by using the SuperScript® One-Cycle cDNA Kit, the Nextera XT DNA Library Preparation Kit and V3 chemistry (2 × 300 bp) according to the manufacturers’ instructions. After normalization, all samples were pooled and sequenced on an Illumina MiSeq instrument, with the aim of ~25 million pair-end reads. In total, 19.5 million high quality sequence reads were obtained from 12 pools, averaging 1.5 million reads per pool. NGS data of all pools were analysed together by using DIAMOND^81^ against the whole non-redundant virus protein database (NCBI Ref Seq as of 1^st^ February 2018). No significant hits were found against viral taxa other than Adenoviruses. All NGS reads successfully identified as AdVs and related reference sequences from NCBI Genbank were used for the development of a Lemur-AdV hemi-nested PCR. Primer sequences targeting the AdV hexon gene were as follows: AdenoV-Fwd; GTG GAC AAR GAA GAY ACC CAG TAC; AdenoV-Fnest, AYC GSG TGC TKG AYA TGG GRA G; AdenoV-Rev, GTC YCK RAA WCC AAT GTA GTT RGG. The PCR was set up containing 5 μl extracted DNA, 400 nM of the respective forward and reverse primers, 10x Platinum Taq-Buffer (Invitrogen, Life Technologies, Karlsruhe, Germany), 200 nM each deoxynucleotide triphosphates, 2.5 mM MgCl_2_ and 0.5 U Invitrogen™ Platinum™ Taq DNA (Invitrogen). The amplification protocol consisted of 3 min at 94 °C, 45 cycles of 15 s at 95 °C, 15 s at 58 °C and 60 s at 68 °C, with a final elongation step of 3 min at 68 °C. The master mix and amplification profile for the 2^nd^ round of PCR were identical to the 1st round PCR, although 1 µl of the 1^st^ round product was used as template. All 2^nd^ round PCR products were visualized by agarose gel electrophoreses with ethidium bromide staining and were Sanger-sequenced (SEQLAB Sequence Laboratories, Göttingen, Germany) to confirm the presence of AdV sequences.

To obtain data about the individual AdV infection status, we screened all 160 samples by using our Lemur-AdV hemi-nested PCR assay as described above. The quantification of AdV DNA was performed using real-time PCRs designed to allow the detection of lemur AdV-sequences (primer and probe sequences are available upon request). The later have been identified by the screening assay and by using photometrically quantified plasmids carrying the PCR amplicons from the initial screening assay^82^. The average AdV DNA concentration in the 44 positive samples was high with 3.6 × 10^9^ DNA copies/mg feces (range 1 × 10^5^ to 9 × 10^10^) suggesting rather acute than latent adenovirus infections in these individuals (Supplementary Table S4). In the microbiome analysis, we included all 44 AdV-positive samples and 116 AdV-negative samples.

### Bacterial DNA extraction and 16S rRNA gene amplicon sequencing

Faecal samples were homogenized by using beads in a SpeedMill PLUS Homogenizer (Analytik Jena, Germany). Bacterial genomic DNA was extracted from faeces by using NucleoSpin 96 Soil kit (Macherey-Nagel, Germany) according to the manufacturer’s instructions. We amplified the hypervariable V4 region of the 16S rRNA gene (291 bp) with the primer pair 515F (5-GTGCCAGCMGCCGCGGTAA-3) and 806R (5-GGACTACHVGGGTWTCTAAT-3)^83,84^. Following the approach of the Fluidigm System (Access Array™ System for Illumina Sequencing Systems, © Fluidigm Corporation), these primers were tagged with sequences (CS1 forward tag and CS2 reverse tag) complementary to the respective forward or reverse access array barcode primers required for the Illumina platform. The PCR mix (15 μl) was composed of 10 ng/μl DNA, 1X FastStart PCR grade nucleotide mix buffer without MgCl_2_ (Roche), 4.5 nM MgCl_2_ (Roche), 200 μM each PCR grade nucleotide (Roche), 0.05 U/μl Fast Start Taq DNA Polymerase (Roche), 400 nM access array barcode primers for Illumina (Fluidigm), 5% DMSO (Roche), 4% PCR certified water and 50 nM target-specific primers (515F and 806R). All individual samples were processed as described in the Fluidigm’s protocol (Access Array®, Fluidigm 2012, San Francisco, USA). Barcoded samples were purified by using NucleoMag bead-based size selection (Macherey-Nagel, Germany) in a robot device (GeneTheatre, Analytik Jena, Germany). After quantification by DropSense (Trinean, US), samples were pooled to give an equal amount of 50 ng DNA and the pool was diluted to 8 pM in hybridization buffer. The final library was paired-end sequenced in a single run on an Illumina^®^ MiSeq platform. Negative controls were included in both the DNA extraction (no sample added) and 16S PCR amplification (with PCR certified water) to test for contamination. No DNA contamination was observed by using DropSense quantification.

After the run, forward and reverse reads were demultiplexed and sequences with corresponding barcodes were merged by using FLASH-1.2.11^85^. Primer sequences together with linkers (i.e. small 4 base-pair random sequences added between primers and fluidigm tags for cluster generation) were removed by using CUTADAPT 1.11^86^. Sequences that were too long or too short were removed from the dataset by using PRINSEQ-lite 0.20.4^87^. We followed the work flow pipeline of the QIIME 1.9.1 software package^88^ for initial quality filtering and further analysis as performed previously^89^. Sequences below the quality threshold (q = 25) or with homopolymers or more than six ambiguous bases were discarded. Potential chimeras were removed by using USEARCH 6.1. Operational Taxonomic Units (OTUs) were picked by using the open-reference OTU picking approach at a 97% similarity threshold with UCLUST as the default (Greengenes database, version 13.8, http://greengenes.lbl.gov). *De novo* OTUs, i.e. reads that did not hit the Greengenes database, were also picked. Taxonomy was assigned by using the Ribosomal Database Project (RDP) classifier. Singletons and OTUs belonging to chloroplast, mitochondria, archaea, eukaryota and unassigned OTUs were further removed from the dataset.

### Alpha and beta diversity analysis

To investigate the effect of AdV infection on gut microbial diversity, we calculated alpha diversity measurements for each sample by using three different diversity indices (number of observed species (OTUs), Chao1 and phylogenetic diversity (PD)) after rarefying the data to 22,200 sequences per sample. All further analyses were done in R version 3.3.2^90^. To analyse the association of AdV infection with these alpha diversity matrices, we performed General Linear Modelling (GLM) by using the *lme*_*4*_ package in R^91^. We included AdV infection (N_AdV−_ = 116, N_AdV+_ = 44), age (young = 61, adult = 85) and interaction between these two parameters (AdV*age) in the model as explanatory variables for each alpha diversity metric.

To investigate the association of the AdV infection with gut microbial beta diversity, we calculated unweighted UniFrac, weighted UniFrac and Bray-Curtis metrics after rarefying the data to 22,200 sequences per sample by using the *phyloseq* package in R^26^. The PERMANOVA approach (*adonis* in R package *vegan*) was used to test the significance of the differences in the community composition with 999 permutations. For all three beta diversity metrics, we included, as previously, AdV infection, age and interaction between these two parameters (AdV*age) in the models as explanatory variables. Furthermore, in order to visualize the pattern of separation between different sample categories, principal coordinates analyses (PCoA) were plotted based on the UniFrac and Bray-Curtis metrics. We used Mann-Whitney-Wilcoxon tests to deduce the AdV effect on the inter-individual variability of the three beta diversity metrics in AdV− and AdV+ individuals. Moreover, we investigated whether AdV− and AdV+ individuals differ in their body mass and whether body mass is associated with bacterial alpha and beta diversity by running GLM and PERMANOVA models, as described above.

In order to understand which bacterial phyla composition were specifically influenced by AdV infections, we calculated unweighted and weighted UniFrac metrics for the five major phyla separately and ran PERMANOVA with 999 permutations including AdV infection, age, and the interaction AdV*age in the models as explanatory variables as previously described.

### Relative abundance of major taxa and identification of OTUs associated with AdV infections

We compared the relative abundance of the five major taxa at phylum and class level by using Mann-Whitney-Wilcoxon tests to understand their association with AdV infections but also with age on higher taxonomic levels. In order to identify the OTUs accountable for differences in the microbiome of AdV− and AdV+ lemurs, we employed a negative binomial model-based approach available in the *edgeR* package in R^92^ after removing spurious OTUs (i.e. OTUs present in less than three samples for each category). Exact tests (Exact binomial test generalized for overdispersed counts) were performed and only OTUs remaining significant (p < 0.01) after the Benjamini–Hochberg correction were reported.

### Microbial network analyses

In order to infer the relationships among OTUs, we prepared microbial correlation networks for each AdV− and AdV+ category by using CoNet plugin for Cytoscape^93^. To reduce the network complexity and remove rare OTUs, we restricted our analyses to OTUs present in at least 25% of the samples for each AdV− and AdV+ category. We employed Spearman correlation and Bray-Curtis dissimilarity to calculate all pairwise taxa (OTU) scores.1000 positive and 1000 negative edges were selected as the threshold for the initial network and for each measure and each edge, 100 renormalized permutation and bootstraps scores were generated^93^. Measure-specific P-values were merged by using Brown’s method and, after correction for multiple testing following the Benjamini–Hochberg procedure, only stable edges with merged P-values below 0.05 were retained.

Additionally, we prepared a correlation network for OTUs which were significantly associated with AdV-infections. We restricted these analyses to those OTUs that differed in their relative abundance in AdV− and AdV+ lemurs as identified by the Exact-test approach (78 OTUs, see above). Here, to calculate all pairwise taxa (OTU) scores, we applied an ensemble approach by using five similarity measures (‘Pearson correlation’, ‘Spearman correlation’, ‘Bray-Curtis dissimilarity’, ‘Kullback-Leibler dissimilarity’ and ‘mutual information’) established as being more precise than the use of a single correlation detection strategy, especially for sparse data, as in the case of network with limited 78 OTUs^94^. Similar network building parameters were used as described above.

### Ethics approval

This research was approved by the ethics committee of the Institute of Zoology of Hamburg University, the University of Antananarivo and Madagascar National Parks. Permission to conduct the use of these animals and the collection of faecal samples was granted by the Autorisation de Recherche No. 54/13/MEF/SG/DGF/DCB.SAP/SCB of February 22, 2013, issued by the Direction Générale des Forêts and the Direction de la Conservation de la Biodiversité et du Système des Aires Protégées of the Ministère de l’Environnement, et des Forêts and exported to Germany under the CITES permit 576C-EA09/MG14. We confirm that all methods were performed in accordance with the relevant guidelines and regulations.

## Supplementary information

Supplementary Information

## Data Availability

The individual gut bacterial 16S rRNA gene sequences are available under SRA accession ID SRP217185.
