# Supplementary material for: Adenovirus infection is associated with altered gut microbial communities in a non-human primate
Source: Sci Rep. 2019 Sep 16;9:13410. doi: 10.1038/s41598-019-49829-z (PMC6746978; doi:10.1038/s41598-019-49829-z)
Supplement: Supplementary file 1 — Supplementary Information [file 41598_2019_49829_MOESM1_ESM.pdf]

Supplementary information

Adenovirus infection is associated with altered gut microbial communities in a non-human primate

Wasimuddin, Victor M. Corman, Jörg U. Ganzhorn, Jacques Rakotondranary, Yedidya R. Ratovonamana, Christian Drosten, Simone Sommer

Supplementary Figure 1

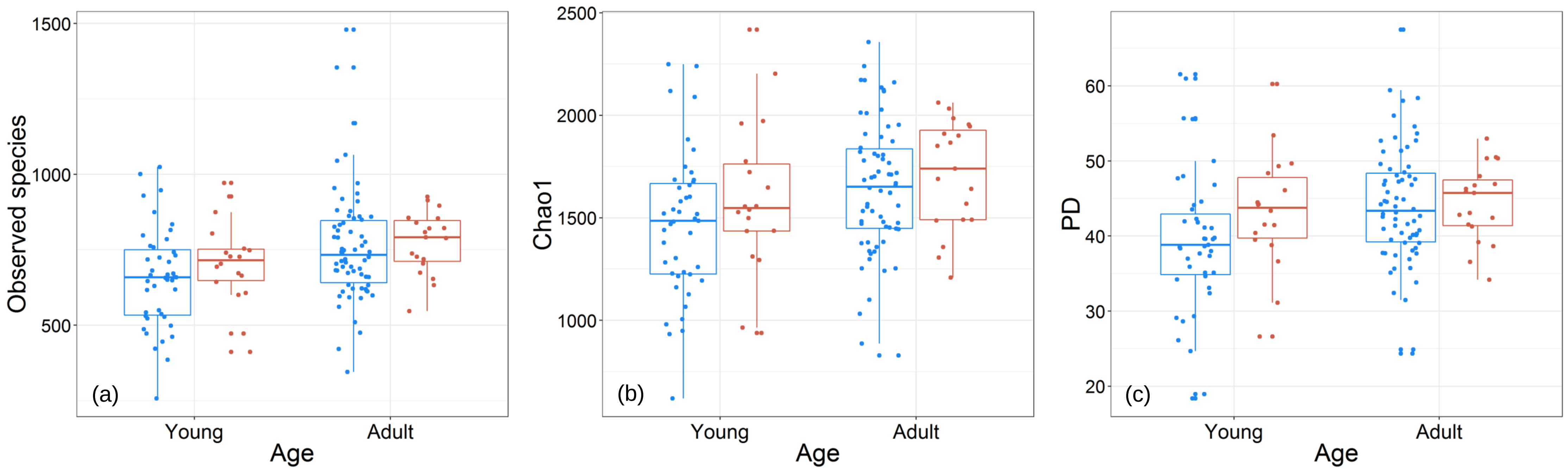

Supplementary Figure 1. Gut bacterial alpha diversity according to AdV infection and age in mouse lemurs. Shown is the effect of AdV infection (blue; AdV-, red; AdV+) and age on (a) Number of observed species (p=0.722), (p=0.002), (b) Chao1 (p=0.465), (p=0.007) and (c) PD (p=0.880), (p=0.003) on mouse lemur individuals, respectively (see Supplementary Table 1 for details).

Supplementary Figure 2

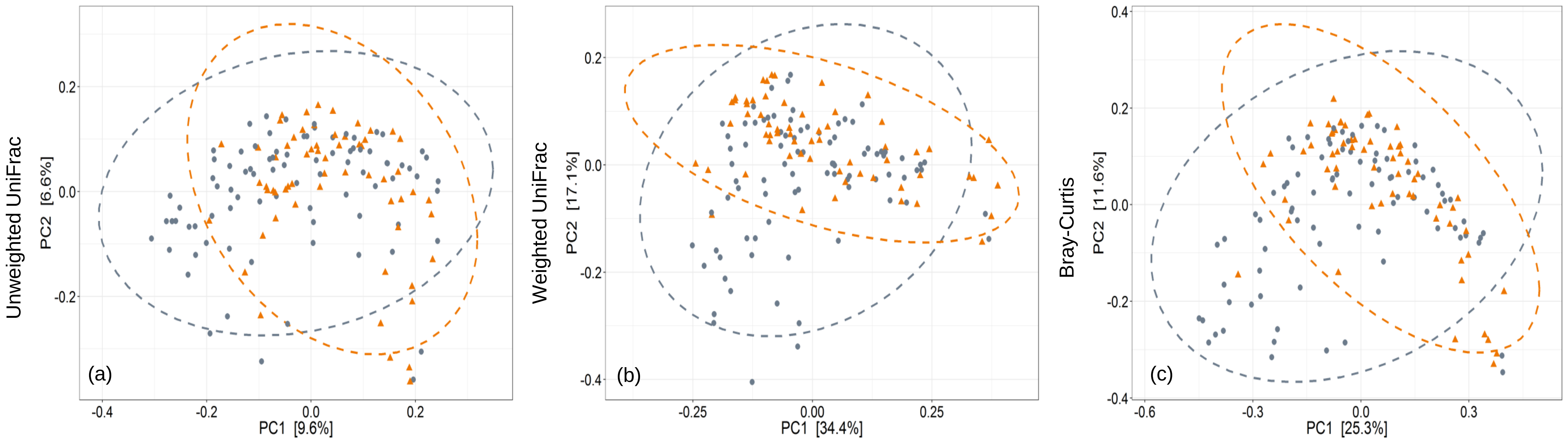

Supplementary Figure 2. Gut bacterial community composition of mouse lemurs according to age. Principal-coordinate plots based on (a) unweighted UniFrac (p=0.001), (b) weighted UniFrac (p=0.005) and (c) Bray-Curtis (p=0.001) metrics of gut bacterial communities of mouse lemurs according to age. Dots/triangles and surrounding dashed ellipses (95% confidence level) represent gut bacterial communities of young (orange) and adults (grey).

Supplementary Figure 3

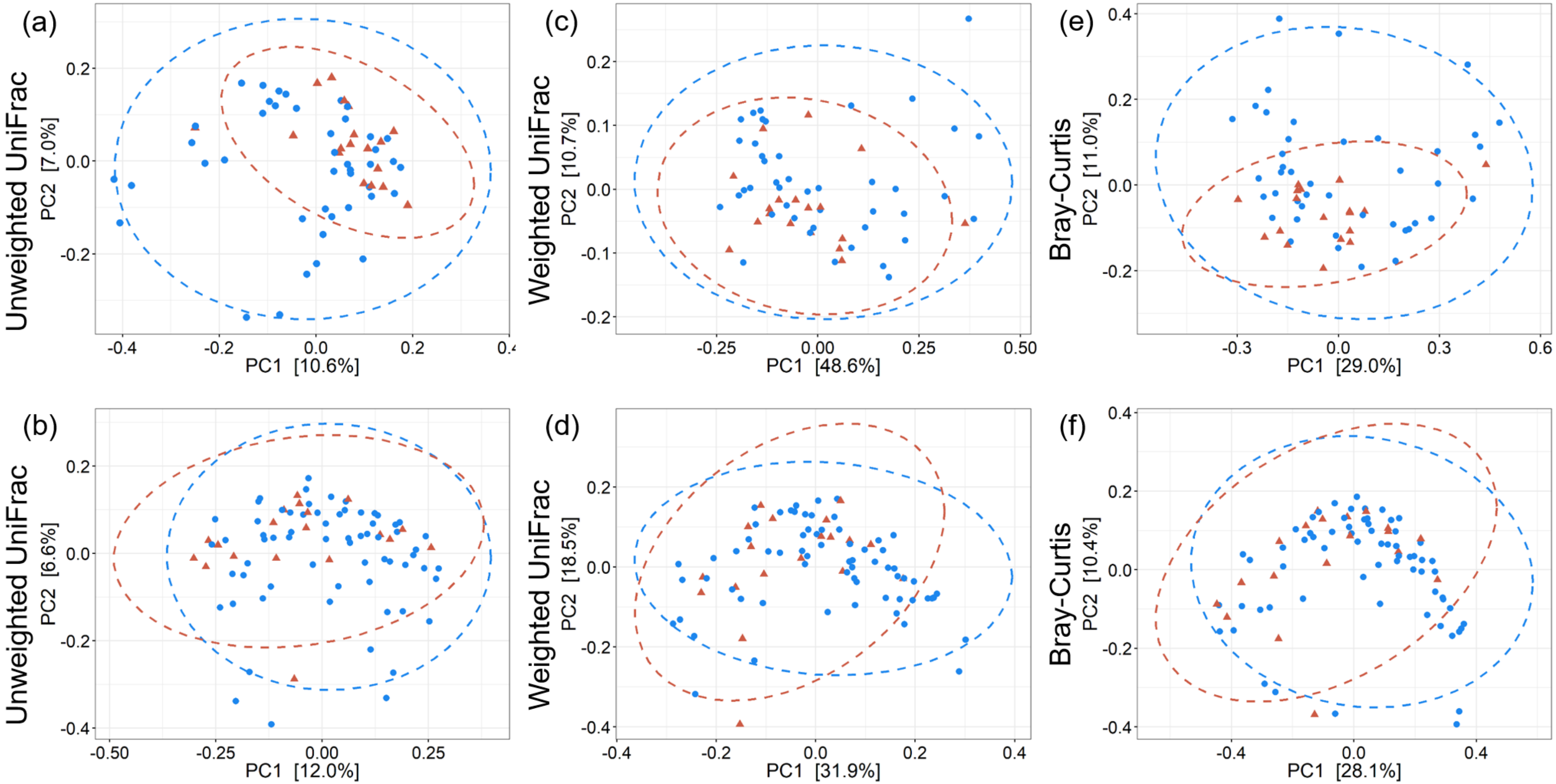

Supplementary Figure 3. Gut bacterial community compositions according to AdV infection in young and adult mouse lemurs. Principal-coordinate plots based on (a, b) unweighted UniFrac (AdV*Age, p=0.172), (c, d) weighted UniFrac (AdV*Age, p=0.704) and (e, f) Bray-Curtis (AdV*Age, p=0.282) metrics of gut bacterial communities of young (a, c, e) and adult (b, d, e) mouse lemurs. Dots/triangles and surrounding dashed ellipses (95% confidence level) represent gut bacterial communities of AdV- (blue) and AdV+ (red) individuals.

Supplementary Figure 4

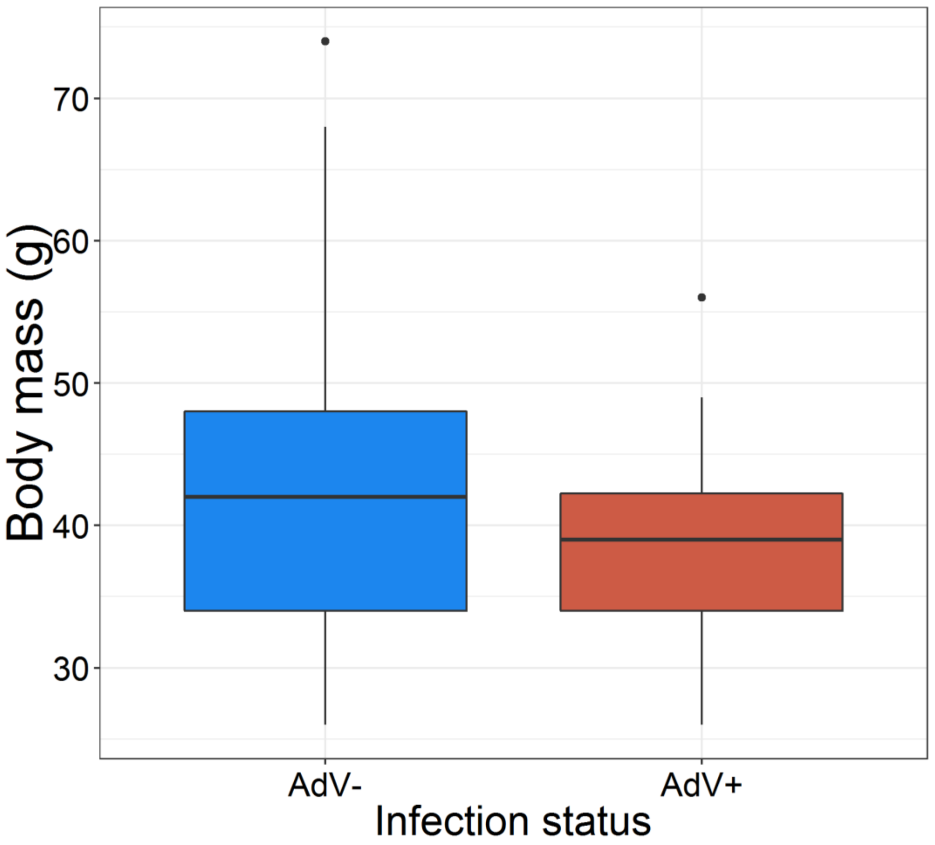

Supplementary Figure 4. Average body mass of AdV- and AdV+ mouse lemurs. The average body mass (in gram) of AdV+ individuals tended to be lower than of AdV- individuals (p=0.062).

Supplementary Figure 5

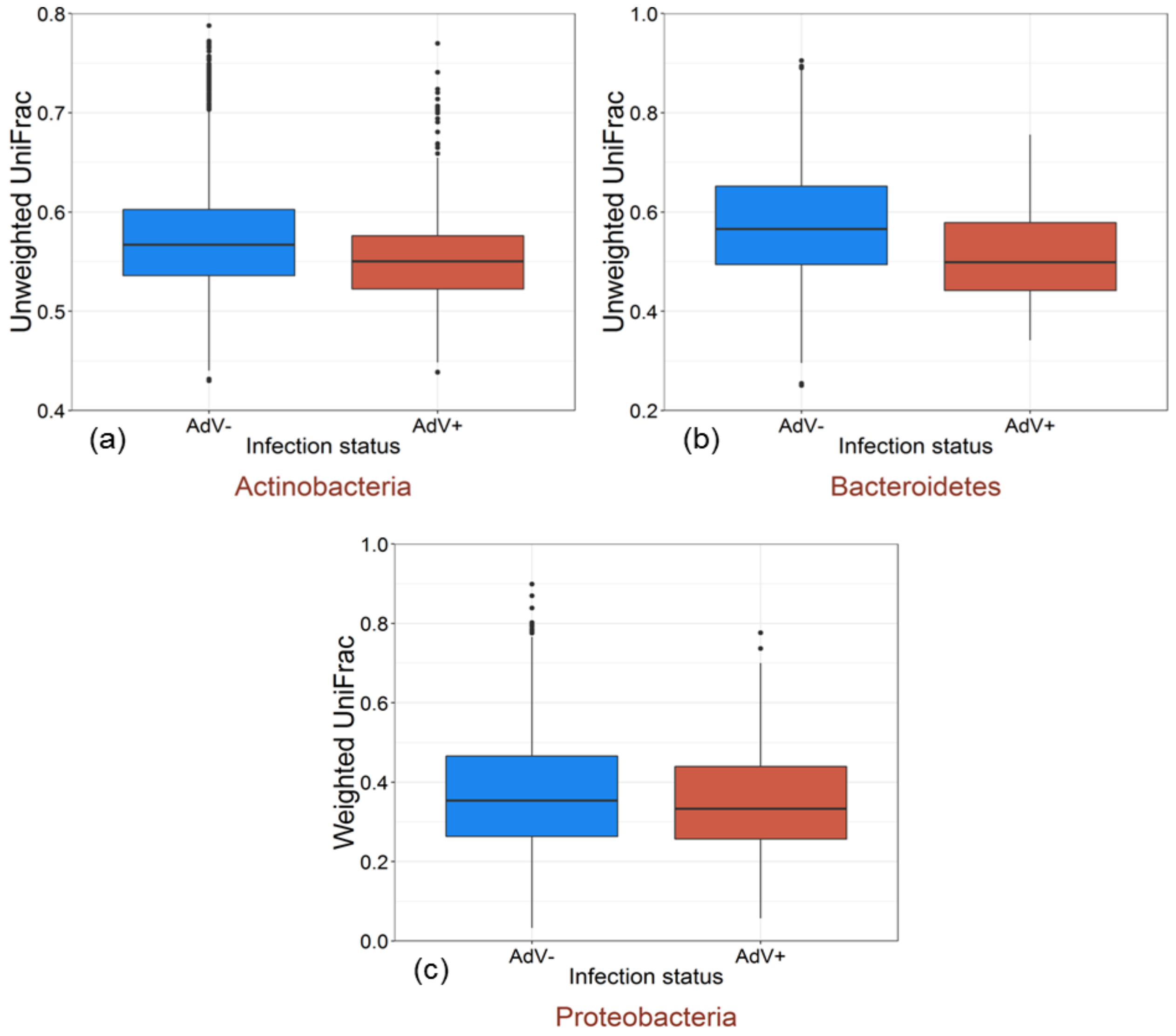

Supplementary Figure 5. Beta diversity metrics of major phyla in relation to AdV infection in mouse lemurs. Boxplots are showing unweighted UniFrac distances in (a) Actinobacteria (34.4% prevalence) (p=0.016) and (b) Bacteroidetes (28.4% prevalence) (p=0.035), and weighted UniFrac distances in (c) Proteobacteria (9.2% prevalence) (p=0.016) in AdV- (blue) and AdV+ (red) mouse lemurs.

Supplementary Figure 6

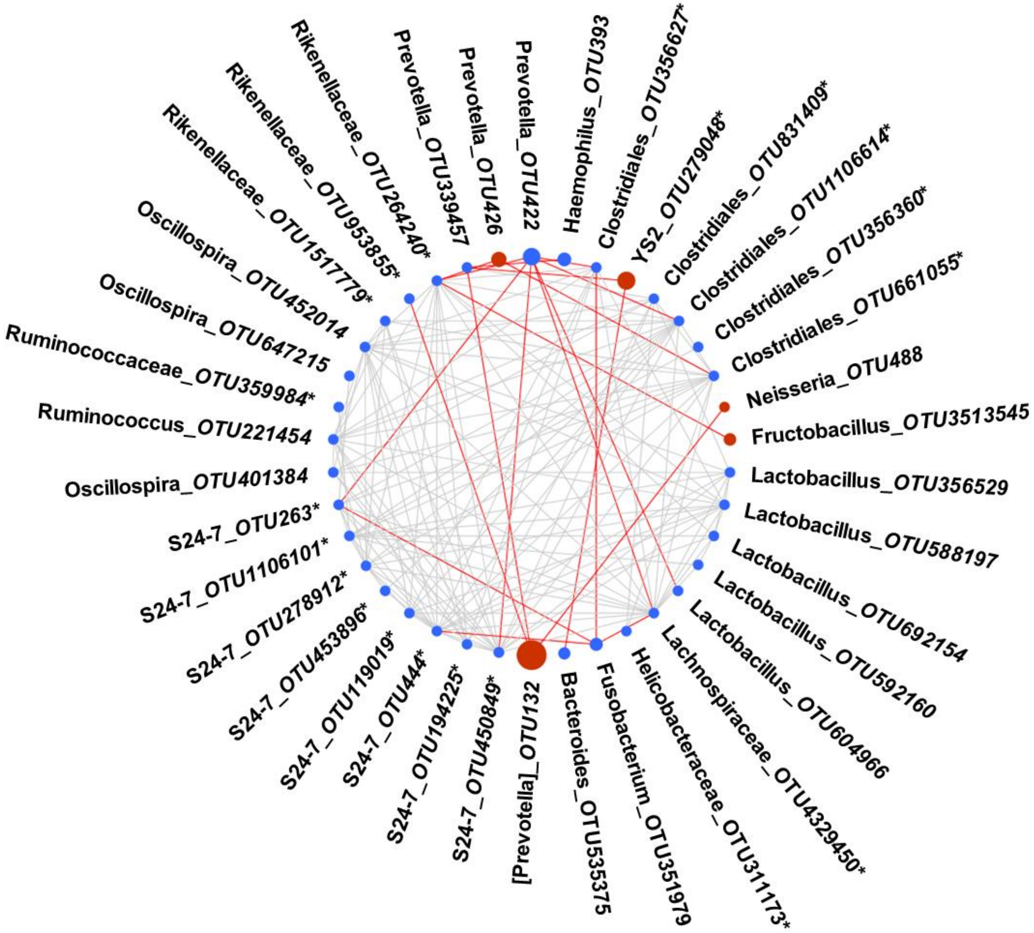

Supplementary Figure 6. Microbial networks of OTUs differing in their relative abundance in relation to AdV-infection in mouse lemurs. Shown is the network of mouse lemurs based on identified OTUs, differing in their relative abundance between AdV infected and uninfected mouse lemurs (sub bipartite and tripartite networks are not shown) (see Supplementary Table S3 for more details). Blue nodes mark decreasing OTUs and red nodes increasing OTUs. Node sizes are according to the relative proportion of the OTUs. Red edges indicate negative relationships and grey edges positive relationships between the interacting OTUs. The highest possible taxonomic assignment (maximal to the genus level) is shown for each OTU. *****indicates unclassified OTUs at genus level.

Supplementary Table S1: Summary of the best model (GLM) predicting (a) observed species, (b) Chao1, and (c) phylogenetic diversity (PD) according to AdV, age and AdV*age interaction. Significant p values (<0.05) are shown in bold.

Variables	Estimate	Std.Error	t value	P
(a) Observed species				
AdV	15.500	43.500	0.356	0.722
Age	-101.470	32.740	-3.099	**0.002**
AdV*age	35.150	63.970	0.549	0.584
(b) Chao1				
AdV	63.580	86.750	0.733	0.465
Age	-179.530	65.300	-2.749	**0.007**
AdV*age	66.880	127.570	0.524	0.601
(c) PD				
AdV	0.317	2.089	0.152	0.880
Age	-4.839	1.572	-3.078	**0.003**
AdV*age	3.742	3.072	1.218	0.225

Supplementary Table S2: Summary of PERMANOVA models of (a) Unweighted UniFrac, (b) Weighted UniFrac and (c) Bray-Curtis metrics according to AdV infection, age and AdV*age interaction. Significant p values (≤0.05) are shown in bold.

Variables	SumsOfSqs	MeanSqs	F.Model	R2	P
(a) Unweighted UniFrac					
AdV	0.292	0.292	1.455	0.010	**0.016**
Age	0.489	0.489	2.440	0.017	**0.001**
AdV*age	0.229	0.229	1.144	0.008	0.172
Residuals	28.454	0.200		0.966	
Total	29.464			1.000	
(b) Weighted UniFrac					
AdV	0.125	0.125	1.955	0.013	**0.055**
Age	0.293	0.293	4.585	0.031	**0.005**
AdV*age	0.042	0.042	0.658	0.004	0.704
Residuals	9.085	0.064		0.952	
Total	9.546			1.000	
(c) Bray-Curtis					
AdV	0.304	0.304	1.870	0.013	**0.037**
Age	0.707	0.707	4.343	0.029	**0.001**
AdV*age	0.188	0.188	1.154	0.008	0.282
Residuals	23.105	0.163		0.951	
Total	24.304			1.000	

Supplementary Table S3: Nomenclature and abundances of 78 OTUs identified by Exact tests in mouse lemurs as differing in their mean abundance in relation to their AdV infection status. Only highly significant OTUs (after FDR correction using Benjamini-Hochberg procedure (BH), ≤0.01) are shown and arranged according to increasing log2 fold change (log2FC) values. In the column log2FC, OTUs that showed a lower abundance (i.e. mean read coverage) in AdV+ lemurs are marked in blue (65.3%), those that increased in abundance in AdV+ lemurs are marked in red (34.6%). Indicated are also the fraction of samples (AdV-, AdV+) in which OTUs are present (in %) and the abundance (number of reads) of an OTU in both AdV- and AdV+ mouse lemurs. samples. Potential pathogenic genera are marked in red (see Fig. 3).

Page1

OTU	AdV- fraction (%)	AdV+ fraction (%)	AdV- reads	AdV+ reads	log2FC	PValue	BH
OTU450849	3.67	0	4081	0	-5,602	1,12E-11	2,11E-10
OTU354254	2.75	2.7	3093	1	-4,746	2,19E-10	3,25E-09
OTU339457	1.83	2.7	1881	1	-4,625	2,78E-10	3,85E-09
OTU583656	13.76	13.51	2676	12	-4,400	2,19E-10	3,25E-09
OTU661055	2.75	0	1362	0	-4,181	9,77E-10	1,26E-08
OTU264240	3.67	0	1485	0	-4,096	1,22E-09	1,41E-08
OTU1106614	2.75	2.7	1289	1	-3,950	2,15E-09	2,23E-08
OTU564704	56.88	51.35	16981	292	-3,861	5,22E-07	2,31E-06
OTU452014	3.67	2.7	1124	1	-3,828	3,52E-09	3,35E-08
OTU276149	10.09	8.11	1467	3	-3,819	1,52E-09	1,66E-08
OTU692154	4.59	2.7	1442	1	-3,801	3,55E-09	3,35E-08
OTU178491	2.75	2.7	1387	1	-3,603	1,07E-08	7,95E-08
OTU4329450	2.75	0	1035	0	-3,546	9,16E-09	7,95E-08
OTU444	2.75	0	968	0	-3,521	9,94E-09	7,95E-08
OTU221454	4.59	0	864	0	-3,512	1,00E-08	7,95E-08
OTU263	2.75	0	909	0	-3,508	9,26E-09	7,95E-08
OTU356627	1.83	0	934	0	-3,462	1,39E-08	9,96E-08
OTU647215	6.42	5.41	865	2	-3,435	1,03E-08	7,95E-08
OTU311173	1.83	2.7	776	1	-3,402	2,21E-08	1,48E-07
OTU535375	27.52	21.62	5445	153	-3,372	9,27E-07	3,78E-06
OTU604966	8.26	0	931	0	-3,321	1,62E-08	1,13E-07
OTU592160	3.67	2.7	978	1	-3,248	3,22E-08	2,03E-07
OTU1107027	5.5	0	923	0	-3,221	2,71E-08	1,76E-07

OTU422	21.1	10.81	9406	480	-3,146	1,32E-05	4,56E-05
OTU356529	3.67	0	813	0	-3,109	4,74E-08	2,90E-07
OTU194225	2.75	0	593	0	-3,017	9,06E-08	5,39E-07
OTU1517779	3.67	0	576	0	-2,969	9,71E-08	5,61E-07
OTU116901	3.67	0	479	0	-2,829	2,48E-07	1,25E-06
OTU453896	3.67	0	568	0	-2,817	1,77E-07	9,68E-07
OTU119019	3.67	0	570	0	-2,817	1,90E-07	1,01E-06
OTU831409	3.67	0	471	0	-2,791	2,53E-07	1,25E-06
OTU671	22.94	45.95	3824	166	-2,782	2,40E-05	8,19E-05
OTU278912	2.75	0	506	0	-2,767	2,41E-07	1,25E-06
OTU208843	5.5	5.41	575	4	-2,723	2,76E-07	1,33E-06
OTU401384	3.67	2.7	439	1	-2,659	4,86E-07	2,25E-06
OTU356360	2.75	2.7	618	1	-2,640	4,65E-07	2,20E-06
OTU228061	10.09	8.11	1189	57	-2,623	7,03E-06	2,61E-05
OTU351979	22.94	13.51	8104	522	-2,620	0,000338109	0,001049652
OTU271101	1.83	0	394	0	-2,588	8,15E-07	3,39E-06
OTU953855	5.5	2.7	802	21	-2,559	3,63E-06	1,37E-05
OTU588197	4.59	0	486	0	-2,553	6,50E-07	2,82E-06
OTU1106101	2.75	2.7	478	1	-2,367	1,55E-06	6,18E-06
OTU359984	4.59	2.7	450	1	-2,297	2,03E-06	7,95E-06
OTU320915	4.59	8.11	668	15	-2,256	9,86E-06	3,60E-05
OTU1111294	62.39	72.97	2872	197	-2,152	0,000110518	0,000364885
OTU583117	22.02	21.62	5051	381	-2,143	0,001063886	0,003073449
OTU393	61.47	72.97	3792	330	-1,961	0,00027432	0,000864523
OTU4456606	24.77	32.43	1076	81	-1,833	0,000237586	0,000760277
OTU364179	11.01	8.11	1549	179	-1,832	0,002542556	0,006868202
OTU208479	10.09	5.41	583	46	-1,537	0,00119764	0,003412454
OTU228	99.08	97.3	15610	2637	-1,207	0,000630084	0,001845879
OTU317	99.08	100	8469	6087	1,001	0,00157424	0,004424892
OTU132	80.73	94.59	17612	14122	1,180	0,002167545	0,005932229
OTU591635	23.85	35.14	1260	1043	1,264	0,003188812	0,0085035
OTU854050	23.85	32.43	575	596	1,498	0,00036854	0,00111096
OTU426	37.61	51.35	4337	4462	1,518	0,001674689	0,00464447

Page 3

OTU279048	42.2	70.27	7285	7003	1,707	0,000482455	0,001433581
OTU3513545	22.02	29.73	2402	3110	1,836	0,000206611	0,000671486
OTU1106254	3.67	8.11	706	1028	1,843	7,91E-05	0,00026552
OTU325530	11.01	27.03	433	824	1,850	3,47E-06	1,34E-05
OTU528421	20.18	13.51	829	1002	2,041	1,23E-05	4,40E-05
OTU1084865	82.57	86.49	44690	75527	2,171	0,000357862	0,001094638
OTU201	27.52	37.84	4320	7338	2,279	1,28E-05	4,50E-05
OTU336708	21.1	35.14	1601	3168	2,387	7,71E-07	3,27E-06
OTU66	53.21	56.76	3067	6369	2,538	1,14E-07	6,41E-07
OTU179	21.1	37.84	2513	5476	2,557	4,99E-07	2,26E-06
OTU1065569	47.71	40.54	505	1031	2,622	7,44E-11	1,29E-09
OTU766563	13.76	27.03	191	508	2,666	2,34E-13	4,86E-12
OTU938948	33.94	29.73	147	540	2,962	4,25E-18	1,26E-16
OTU135	44.04	40.54	291	987	3,164	1,20E-17	2,78E-16
OTU488	21.1	21.62	70	458	3,179	4,62E-21	1,60E-19
OTU254021	24.77	40.54	3155	8156	3,248	1,03E-09	1,26E-08
OTU818603	15.6	18.92	172	797	3,270	9,79E-18	2,55E-16
OTU988314	21.1	24.32	109	739	3,618	6,32E-24	2,63E-22
OTU1084417	40.37	37.84	194	1194	3,834	1,54E-24	8,01E-23
OTU849156	8.26	13.51	15	525	3,876	3,20E-31	3,33E-29
OTU1097359	9.17	13.51	96	1783	4,954	2,34E-35	4,87E-33
OTU761024	49.54	56.76	513	5379	4,962	1,67E-29	1,16E-27

Page1 continued

Kingdom	Phylum	Class	Order	Family	Genus	Species
Bacteria	Bacteroidetes	Bacteroidia	Bacteroidales	S24-7	NA	NA
Bacteria	Firmicutes	Clostridia	Clostridiales	Lachnospiraceae	Coprococcus	NA
Bacteria	Bacteroidetes	Bacteroidia	Bacteroidales	Prevotellaceae	Prevotella	copri
Bacteria	Bacteroidetes	Bacteroidia	Bacteroidales	Bacteroidaceae	Bacteroides	NA
Bacteria	Firmicutes	Clostridia	Clostridiales	NA	NA	NA
Bacteria	Bacteroidetes	Bacteroidia	Bacteroidales	Rikenellaceae	NA	NA
Bacteria	Firmicutes	Clostridia	Clostridiales	NA	NA	NA
Bacteria	Firmicutes	Bacilli	Lactobacillales	Leuconostocaceae	NA	NA
Bacteria	Firmicutes	Clostridia	Clostridiales	Ruminococcaceae	Oscillospira	NA
Bacteria	Bacteroidetes	Bacteroidia	Bacteroidales	Porphyromonadaceae	Parabacteroides	NA
Bacteria	Firmicutes	Bacilli	Lactobacillales	Lactobacillaceae	Lactobacillus	reuteri
Bacteria	Proteobacteria	Alphaproteobacteria	RF32	NA	NA	NA
Bacteria	Firmicutes	Clostridia	Clostridiales	Lachnospiraceae	NA	NA
Bacteria	Bacteroidetes	Bacteroidia	Bacteroidales	S24-7	NA	NA
Bacteria	Firmicutes	Clostridia	Clostridiales	Ruminococcaceae	Ruminococcus	NA
Bacteria	Bacteroidetes	Bacteroidia	Bacteroidales	S24-7	NA	NA
Bacteria	Firmicutes	Clostridia	Clostridiales	NA	NA	NA
Bacteria	Firmicutes	Clostridia	Clostridiales	Ruminococcaceae	Oscillospira	NA
Bacteria	Proteobacteria	Epsilonproteobacteria	Campylobacterales	Helicobacteraceae	NA	NA
Bacteria	Bacteroidetes	Bacteroidia	Bacteroidales	Bacteroidaceae	Bacteroides	ovatus
Bacteria	Firmicutes	Bacilli	Lactobacillales	Lactobacillaceae	Lactobacillus	NA
Bacteria	Firmicutes	Bacilli	Lactobacillales	Lactobacillaceae	Lactobacillus	NA
Bacteria	Firmicutes	Bacilli	Lactobacillales	Lactobacillaceae	Lactobacillus	NA

Page2 continued

Bacteria	Bacteroidetes	Bacteroidia	Bacteroidales	Prevotellaceae	Prevotella	NA
Bacteria	Firmicutes	Bacilli	Lactobacillales	Lactobacillaceae	Lactobacillus	NA
Bacteria	Bacteroidetes	Bacteroidia	Bacteroidales	S24-7	NA	NA
Bacteria	Bacteroidetes	Bacteroidia	Bacteroidales	Rikenellaceae	NA	NA
Bacteria	Bacteroidetes	Bacteroidia	Bacteroidales	[Odoribacteraceae]	Odoribacter	NA
Bacteria	Bacteroidetes	Bacteroidia	Bacteroidales	S24-7	NA	NA
Bacteria	Bacteroidetes	Bacteroidia	Bacteroidales	S24-7	NA	NA
Bacteria	Firmicutes	Clostridia	Clostridiales	NA	NA	NA
Bacteria	Tenericutes	Mollicutes	Mycoplasmatales	Mycoplasmataceae	NA	NA
Bacteria	Bacteroidetes	Bacteroidia	Bacteroidales	S24-7	NA	NA
Bacteria	Bacteroidetes	Bacteroidia	Bacteroidales	[Odoribacteraceae]	Odoribacter	NA
Bacteria	Firmicutes	Clostridia	Clostridiales	Ruminococcaceae	Oscillospira	NA
Bacteria	Firmicutes	Clostridia	Clostridiales	NA	NA	NA
Bacteria	Firmicutes	Clostridia	Clostridiales	Ruminococcaceae	NA	NA
Bacteria	Fusobacteria	Fusobacteriia	Fusobacteriales	Fusobacteriaceae	Fusobacterium	NA
Bacteria	Bacteroidetes	Bacteroidia	Bacteroidales	S24-7	NA	NA
Bacteria	Bacteroidetes	Bacteroidia	Bacteroidales	Rikenellaceae	NA	NA
Bacteria	Firmicutes	Bacilli	Lactobacillales	Lactobacillaceae	Lactobacillus	reuteri
Bacteria	Bacteroidetes	Bacteroidia	Bacteroidales	S24-7	NA	NA
Bacteria	Firmicutes	Clostridia	Clostridiales	Ruminococcaceae	NA	NA
Bacteria	Cyanobacteria	4C0d-2	YS2	NA	NA	NA
Bacteria	Proteobacteria	Gammaproteobacteria	Enterobacteriales	Enterobacteriaceae	NA	NA
Bacteria	Bacteroidetes	Bacteroidia	Bacteroidales	Bacteroidaceae	Bacteroides	ovatus
Bacteria	Proteobacteria	Gammaproteobacteria	Pasteurellales	Pasteurellaceae	Haemophilus	NA
Bacteria	Cyanobacteria	4C0d-2	YS2	NA	NA	NA
Bacteria	Bacteroidetes	Bacteroidia	Bacteroidales	Bacteroidaceae	Bacteroides	NA
Bacteria	Bacteroidetes	Bacteroidia	Bacteroidales	[Odoribacteraceae]	Butyricimonas	NA
Bacteria	Actinobacteria	Coriobacteriia	Coriobacteriales	Coriobacteriaceae	NA	NA
Bacteria	Actinobacteria	Coriobacteriia	Coriobacteriales	Coriobacteriaceae	Collinsella	NA
Bacteria	Bacteroidetes	Bacteroidia	Bacteroidales	[Paraprevotellaceae]	[Prevotella]	NA
Bacteria	Firmicutes	Clostridia	Clostridiales	Ruminococcaceae	Ruminococcus	NA
Bacteria	Firmicutes	Bacilli	Bacillales	Planococcaceae	NA	NA
Bacteria	Bacteroidetes	Bacteroidia	Bacteroidales	Prevotellaceae	Prevotella	NA

Page3 continued

Bacteria	Cyanobacteria	4C0d-2	YS2	NA	NA	NA
Bacteria	Firmicutes	Bacilli	Lactobacillales	Leuconostocaceae	Fructobacillus	NA
Bacteria	Bacteroidetes	Bacteroidia	Bacteroidales	[Paraprevotellaceae]	Paraprevotella	NA
Bacteria	Firmicutes	Erysipelotrichi	Erysipelotrichales	Erysipelotrichaceae	RFN20	NA
Bacteria	Proteobacteria	Gammaproteobacteria	Enterobacteriales	Enterobacteriaceae	Citrobacter	NA
Bacteria	Firmicutes	Bacilli	Bacillales	NA	NA	NA
Bacteria	Bacteroidetes	Bacteroidia	Bacteroidales	Bacteroidaceae	Bacteroides	NA
Bacteria	Firmicutes	Clostridia	Clostridiales	Lachnospiraceae	NA	NA
Bacteria	Firmicutes	Erysipelotrichi	Erysipelotrichales	Erysipelotrichaceae	[Eubacterium]	NA
Bacteria	Firmicutes	Clostridia	Clostridiales	Lachnospiraceae	NA	NA
Bacteria	Firmicutes	Bacilli	Lactobacillales	NA	NA	NA
Bacteria	Firmicutes	Bacilli	Lactobacillales	Leuconostocaceae	Leuconostoc	NA
Bacteria	Fusobacteria	Fusobacteriia	Fusobacteriales	Fusobacteriaceae	Fusobacterium	NA
Bacteria	Firmicutes	Bacilli	Lactobacillales	NA	NA	NA
Bacteria	Proteobacteria	Betaproteobacteria	Neisseriales	Neisseriaceae	Neisseria	NA
Bacteria	Firmicutes	Bacilli	Lactobacillales	Leuconostocaceae	Leuconostoc	NA
Bacteria	Firmicutes	Bacilli	Bacillales	Bacillaceae	NA	NA
Bacteria	Proteobacteria	Gammaproteobacteria	Pseudomonadales	Moraxellaceae	Acinetobacter	NA
Bacteria	Proteobacteria	Betaproteobacteria	Burkholderiales	Burkholderiaceae	Lautropia	NA
Bacteria	Proteobacteria	Betaproteobacteria	Burkholderiales	Oxalobacteraceae	NA	NA
Bacteria	Proteobacteria	Gammaproteobacteria	Pseudomonadales	Moraxellaceae	Acinetobacter	NA
Bacteria	Proteobacteria	Betaproteobacteria	Neisseriales	Neisseriaceae	Neisseria	NA

Supplementary Table S4: Viral load information of AdV positive samples based on real-time PCRs.

SID	AdV status	DNA cp/µl	DNA copies/ml StoolSuspension	Log10 DNA copies/ml StoolSuspension	group (virus load)
58	pos	5	60000	4.78	low
59	pos	5	60000	4.78	low
61	pos	56.5	678000	5.83	low
63	pos	72.2	866400	5.94	low
70	pos	35.3	423600	5.63	low
72	pos	26.6	319200	5.50	low
73	pos	5	60000	4.78	low
76	pos	1330	15960000	7.20	intermediate
80	pos	58.9	706800	5.85	low
180	pos	10300	123600000	8.09	high
192	pos	380	4560000	6.66	intermediate
395	pos	2030	24360000	7.39	intermediate
400	pos	246000	2952000000	9.47	high
405	pos	5	60000	4.78	low
408	pos	603	7236000	6.86	intermediate
438	pos	255	3060000	6.49	intermediate
444	pos	5	60000	4.78	low
446	pos	215000	2580000000	9.41	high
447	pos	241	2892000	6.46	intermediate
449	pos	5	60000	4.78	low
451	pos	5	60000	4.78	low
466	pos	5	60000	4.78	low
481	pos	5	60000	4.78	low
603	pos	62.9	754800	5.88	low
605	pos	20200	242400000	8.38	high
606	pos	4490000	53880000000	10.73	high
607	pos	40.9	490800	5.69	low
608	pos	130	1560000	6.19	intermediate
610	pos	10	120000	5.08	low
613	pos	311	3732000	6.57	intermediate
616	pos	64.6	775200	5.89	low
617	pos	16100	193200000	8.29	high

page2

620	pos	1600	19200000	7.28	intermediate
622	pos	347	4164000	6.62	intermediate
625	pos	18.1	217200	5.34	low
626	pos	83.2	998400	6.00	intermediate
641	pos	1100	13200000	7.12	intermediate
642	pos	45.5	546000	5.74	low
646	pos	1780000	21360000000	10.33	high
647	pos	5	60000	4.78	low
649	pos	5	60000	4.78	low
653	pos	39.7	476400	5.68	low
664	pos	15.3	183600	5.26	low
668	pos	1050000	12600000000	10.10	high
